# Half-Moon Shaped Morel-Lavallée Lesion of the Knee

**DOI:** 10.5334/jbr-btr.837

**Published:** 2015-09-15

**Authors:** M. T. El-Essawy, F. M. Vanhoenacker

**Affiliations:** 1 Department of Radiology, Damietta Oncology Institute, Damietta, Egypt; 2 Department of Radiology, AZ Sint-Maarten Duffel-Mechelen, Belgium; 3 Department of Radiology, Antwerp University Hospital, Edegem, Belgium; 4 Faculty of Medicine and Health sciences, University of Ghent, Ghent, Belgium

A 42-year old male patient presented with marked swelling of the lower legs after direct impact to the calf muscles due to a motor vehicle accident. The lesion on the right side was larger than the contralateral lesion. On clinical examination, a fluctuating mass at the posterolateral aspect of the both knees and lower legs was seen.

Ultrasound showed a well-delineated anechoic collection between the subcutaneous fat and the fascia of the calf muscles. The lesion were compressible with the ultrasound transducer. On the right side, there was extension of the lesion underneath the lateral collateral ligament. On images with compression, a small intralesional echogenic focus was seen (Fig. A, transverse images without and with compression; 2 small white arrows on the image with compression indicate the echogenic focus). A similar but smaller lesion was seen on the left side.

**Figures A–C:**
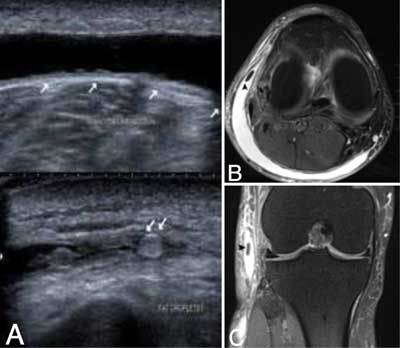


Fat suppressed (FS) T2-weighted (WI) MRI of the right knee revealed a half-moon shaped hyperintense collection between the subcutaneous fat and the fascia of the calf muscle and lateral collateral ligament (Fig. B, axial FS T2-WI). A small focal area of isointense signal compared to subcutaneous fat was seen within the lesion (Fig. B, axial FS T2-WI and Fig. C, coronal FS T2-WI, black arrowheads), in keeping with a fat globule. The diagnosis of Morel-Lavallée lesions was made.

The patient was treated with repeated aspiration of serosanguineous fluid and compression bandage after which the lesions resolved completely.

## Comment

The Morel-Lavallée lesion has been originally described by the French surgeon Victor Auguste Francois Morel-Lavallée in 1863. The mechanism of trauma consists of a degloving injury causing sudden shearing forces at the interface of the superficial fascia and subcutaneous fat. This results in separation of the skin and subcutaneous fatty tissue from the underlying fascia creating a potential space, which may fill by various types of fluid, ranging from serous fluid to frank blood. This serosanguineous collection may either spontaneously resolve or become encapsulated and persistent. The lesion has a predilection for certain location such as the greater trochanter/hip (36%), followed by the thigh (24%) and the pelvis (19%). The knee is the fourth most common involved region (16%).

On ultrasound, the lesion is anechoic or hypo-echoic and has a typical fusiform or oval shape. It is compressible with the ultrasound transducer. Intralesional fat globules may be seen as small hyperechoic foci. Some lesions may contain internal septations or a fluid-fluid level.

Magnetic resonance imaging (MRI) may be used for more precise assessment of the lesion’s extent, particularly in large lesions. It provides the referring clinician with a more global overview and may be useful for evaluation of the shape of the lesion. In our case, the lesion on the right knee has a peculiar semilunar appearance. The signal of the lesion may vary depending on the signal of internal blood degradation products. In most scenario’s, the signal is hypointense or slightly hyperintense on T1-WI and hyperintense on T2-WI. As on ultrasound, fluid-fluid levels, internal septations or fat globules may be present.

Treatment consists of aspiration and compression bandages. In chronic lesions in which a peripheral capsule has formed, resection of this capsule may be required in order to prevent re-accumulation of hemolymphatic fluid.

In conclusion, the clues to the correct diagnosis of a Morel-Lavallée lesion are the history of a closed degloving injury and the typical location of the lesion between the fascia and the subcutaneous fat. Although most lesions are fusiform or oval, a more crescentic shape may be seen in larger lesions, such as in our case.

## Competing Interests

The authors declare that they have no competing interests.
